# The *Healing Encounters and Attitudes Lists* (HEAL): Psychometric Properties of a German Version (HEAL-D) in Comparison With the Original HEAL

**DOI:** 10.3389/fpsyt.2019.00897

**Published:** 2020-01-10

**Authors:** Heike Gerger, Sarah Buergler, Dilan Sezer, Marc Grethler, Jens Gaab, Cosima Locher

**Affiliations:** ^1^Division of Clinical Psychology and Psychotherapy, Faculty of Psychology, University of Basel, Basel, Switzerland; ^2^School of Psychology, University of Plymouth, Plymouth, United Kingdom

**Keywords:** healthcare, non-specific treatment effects, patient-reported measures, German translation, patient attitudes and perceptions

## Abstract

**Introduction:** Over the last years, the interest in understanding health improvements that occur due to non-specific treatment effects, rather than in response to the specific active treatment ingredients, increased. Nevertheless, investigations on patients’ idiosyncratic perspectives on the non-specific aspects of the healing encounter or of the treatment itself that contribute to placebo effects are still rare. The *Healing Encounters and Attitudes Lists* (HEAL) offer a unique and parsimonious set of instruments to measure patients’ views on a variety of non-specific aspects of the caring encounter. The HEAL items can be administered as computerized adaptive tests or short forms that assess the *patient-provider connection*, the *healthcare environment*, *treatment expectancy*, *positive outlook*, *spirituality*, as well as *attitudes towards complementary and alternative medicine*. So far, no German version of the HEAL exists.

**Methods:** The original 168 HEAL items were translated into German (HEAL-D) applying a translation-back-translation procedure. We examined the psychometric properties of HEAL-D in a sample of 165 participants who reported at least one healthcare visit during the last year.

**Results:** The German short forms of HEAL (HEAL-D-SF) showed good internal consistency and test-retest reliability. The factor structure observed in the English original items showed low to moderate model fit in our sample.

**Discussion:** The development of a German version of HEAL in addition to the original English items offers new possibilities for investigating patients’ idiosyncratic perspectives on the non-specific aspects of treatments across language borders. We will close with presenting possible clinical application as well as promising and relevant future research directions using HEAL-D-SF, including for instance large-scale, cross-national investigations.

## Introduction

Different aspects of healthcare interventions and of the healing encounter itself may influence health outcomes and well-being of patients. Typically, these aspects have been classified into two groups: First, certain treatment components are deduced from specific treatment theories, and have been referred to as characteristic, active, or (disorder-)specific treatment components (1). They are assumed to actively and directly affect health and symptom improvement (e.g. pharmacological ingredients in medications, particular exercises in physiotherapy, or the confrontation with a feared stimulus in exposure-based psychotherapy). Second, healthcare interventions typically take place in a context of care (2) in which additional aspects, such as the therapeutic bond or relationship between a healthcare professional and a patient (3), a plausible rationale for the treatment (4), the treatment providers’ warmth (5, 6) as well as aspects of the treatment setting and environment, impact treatment success (7). These aspects have previously been labelled as non-specific, common, general, incidental, or contextual and their effects are typically described as placebo effects. While there are conceptual differences between the individual labels, all these aspects are assumed to be interacting with the characteristic, active or specific treatment components in contributing to health improvements. In the following we will use the terms specific effects when referring to the first kind of treatment effects and non-specific effects when referring to the latter kind of treatment effects.

In healthcare outcome research, which aims at identifying efficacious active treatments and treatment components, placebos (and other inert treatments) are used to keep all of the non-specific treatment components constant, while manipulating the presence of the specific treatment component. Accordingly, controlling for the non-specific treatment effects in placebo-controlled randomised trials became the gold-standard in healthcare research (8). However, when evaluating more complex treatment packages the realization of a high-quality placebo-controlled study design, intended to control for the non-specific treatment effects, turned out to be a challenge (9–12). In addition the validity of distinguishing between specific and non-specific treatment components has been questioned empirically (13–16), as well as theoretically (17–19).

When turning from the highly controlled setting of health outcome research towards the practice of healthcare, where the actual improvement of a presenting patients’ health is the major goal, several questions regarding the role of the non-specific treatment aspects and their potential effects arise: How relevant are the placebo effects, and thus the effects of the non-specific treatment components? How much do they contribute to patients’ health improvement? Do certain patients benefit more from non-specific treatment components than others? And can non-specific treatment aspects support and boost the effectiveness of a standard treatment (18, 20, 21)?

Recently, an increased interest in understanding and investigating the effects of non-specific treatment aspects can be observed. This research has shown that in addition to the above-mentioned non-specific aspects of the healthcare encounter itself, patients’ perceptions and attitudes are associated with health-related outcomes across diverse healthcare settings. These perceptions and attitudes include patients’ treatment outcome expectations (22–26), patients’ trust in their treatment provider (27), or patients’ spirituality (28, 29). Accordingly, a detailed knowledge about a particular patient’s perception of and attitudes towards certain non-specific treatment aspects might enable treatment providers to specifically tailor the context in which interventions take place as well as the intervention itself to a certain patient’s needs.

The “Healing Encounters and Attitudes Lists” (HEAL) have been developed as a precise and concise set of patient-report measures for assessing attitudes towards and perceptions of several treatment components that are associated with non-specific treatment effects (30). HEAL item banks were constructed following the rigorous instrument development methodology of PROMIS^®^ (31, 32), which combines literature reviews, surveys, clinician interviews, focus groups, cognitive interviews to assess item clarity, exploratory and confirmatory factor analyses, and item response theory methods. The convergent and discriminant validity of the initial items was demonstrated in two samples with over 1600 participants (30). The final item banks include a total of 168 Items reflecting six scales: patient-provider connection (57 items, e.g., *I trust my healthcare provider*), healthcare environment (25 items, e.g., *My care was well organized*.), positive outlook (27 items, e.g., *I am hopeful about my future*.), treatment expectancy (27 items, e.g., *I expect good outcomes of this treatment*.), spirituality (26 items, e.g., *Spiritual beliefs give me hope*.), and attitude toward complementary and alternative medicine (CAM; 6 items, e.g., *I prefer natural remedies*.). Participants are asked to rate items in relation to their current treatment on a five-point Likert scale (*never*, *rarely*, *sometimes*, *often*, and *almost always*). The items are generally applicable in clinical practice, and are not restricted to any type of treatment modality. The HEAL scales are independent of one another: researchers or clinicians can choose which HEAL scales to use. HEAL scales can also be administered as computerized adaptive tests. In computerized adaptive testing the test will be adapted individually to the test-takers responses. If the HEAL items were administered as computerized adaptive tests not all items belonging to one scale would be administered, but based upon the respondent’ previous answers the following items would dynamically be selected for administration.

Short forms of the HEAL (HEAL-SF) have been proposed, with seven items for patient-provider-connection, and six items for healthcare environment, positive outlook, treatment expectancy, spirituality, and attitude toward CAM, respectively (30). Clinical experts selected items for the short forms that had excellent psychometric properties and that were considered to represent the clinical range of each scale of items. The HEAL-SF demonstrated excellent internal consistency which ranged between 0.92 and 0.97.

For clinical practice, particularly the HEAL-SF scales may be applied as a parsimonious assessment tool for complementing the treatment process. Certainly the use of HEAL items are not to replace the necessary exchange between a healthcare provider and the patient regarding the patient’s idiosyncratic perceptions of and attitudes towards the treatment. Rather, HEAL item responses provide a formalized assessment about a certain patient’s attitudes towards a number of non-specific treatment aspects, which may result in shared reflections about the treatment implementation, and may inform about necessary adaptions of the treatment in order to meet the patient’s needs.

So far, no comparable item banks in German were available, that assessed patients’ perceptions of and attitudes towards non-specific treatment components that contribute to placebo effects. Therefore, we translated the English version of the HEAL item banks into a German version of HEAL (i.e., HEAL-D). The aim of this study was to investigate the psychometric properties of HEAL-D, with a specific focus on the short versions (HEAL-D-SF) as these have the most potential to being used in clinical practice as well as in research.

## Materials and Methods

### Translation

We translated the HEAL item banks by means of a translation-back-translation procedure in line with the guidelines proposed by Beaton and colleagues (33). First, the original 168 HEAL items were translated into German independently by two translators (MG and a student research assistant) without adding words or introducing new expressions, and a team of the two independent translators and two supervisors (HG and CL) consented on one German version of the HEAL items. Second, this version was translated back into English language by two independent translators (DS and a research assistant), and again a team including the two independent translators and two supervisors (HG and CL) compared the English back translations with the original HEAL items. If both back-translated versions indicated meaningful deviations from the original HEAL items, adjustments in the German wording were applied until a consensus was reached within the team of translators and supervisors.

### Sample

We tested the German version of the HEAL-D items in a sample of 165 subjects who were recruited *via* an internet survey service of the University of Basel (baps.sona-systems.com). Subjects who received healthcare treatments within the past year, aged over 18, were fluent in reading and speaking German, and not under the acute influence of psychoactive drugs were invited to participate in the online survey.

The Local Ethics Committee Ethikkommission Nordwest- und Zentralschweiz, Switzerland, approved the design and informed consent of the study. The database project and the server were coordinated and located at the Division of Clinical Psychology and Psychotherapy of the Faculty of Psychology at the University of Basel, Switzerland.

### Measures

#### Demographic Variables

Demographic variables such as age, gender, mother tongue, and education were initially assessed.

#### Health-Related Questions

Our sample consisted of subjects who have been receiving at least one healthcare treatment within the past year. We assessed health-related characteristics of the sample, such as information regarding the main diagnosis, the according treatment, the practitioner providing the treatment, as well as the place where the treatment was delivered. We asked our participants to refer to the same treatment context in the first and second assessment.

#### Healing Encounters and Attitudes Lists—German Version (HEAL-D)

The HEAL item banks consist of 168 items reflecting six scales: patient-provider connection (PPC; 57 items), healthcare environment (HE; 25 items), positive outlook (PO; 27 items), treatment expectancy (TE; 27 items), spirituality (SP; 26 items), and attitude toward CAM (CAM; 6 items). We used the translated parallel German version (HEAL-D) of the 168 HEAL items. Additionally, we used the German version of the HEAL-SF (30), with seven items for PPC, and six items for HE, PO, TE, SP, and CAM, respectively. The original HEAL-SF scales demonstrated excellent internal consistencies, which ranged between 0.92 and 0.97.

#### Balanced Inventory of Desirable Responding (BIDR)

The short form of the BIDR (34, 35) contains 20 items, 10 of which capture self-deception (BIDR-SD) and 10 of which tap impression management (BIDR-IM). Internal consistencies of the German version of the two subscales ranged between 0.61 and 0.69 across three studies (34).

### Procedure

Recruitment of participants took place online between July and December 2018. The online survey was advertised on markt.unibas.ch, studienteilnahme.ch, a faculty-internal student platform and in various pharmacies in Basel and was open to the public. Students received course credit for their participation.

After giving informed consent, participants were asked to generate a personalized token and were invited to participate in a secure online survey that included demographic and health-related questions as well as standardized questionnaires (including the HEAL-D items, for details see section *Measures*). The items of the standardized questionnaires were presented in a random manner, in order to prevent carry-over effects when answering a relatively large number of items which all belong to one scale (as is the case in the long version of HEAL-D). Participants had to indicate their preference on a 5-point response scale with 0 = *not at all*, 1 = *a little bit*, 2 = *somewhat*, 3 = *quite a bit*, and 4 = *very much*. The online survey was created and conducted in LimeSurvey (36). For the purpose of assessing the retest reliability of the HEAL-D items, participants were invited to complete the survey twice, whereby the median time interval between the first and the second assessment was 31 days (range 20–56). Since participants’ answers were anonymized, the individual tokens allowed us to match the first and second assessments. Participants had to provide their email addresses in the first assessment, so that we were able to contact them 4 weeks later for the second assessment. Afterwards, email addresses were deleted so that the anonymity of the data was guaranteed.

### Statistical Analyses

The major goal of our study was the development of HEAL-D-SF, a parallel version of HEAL-SF in German language. Initially, we excluded those cases from our sample that did not complete at least one entire scale, as well as cases that did not report a current healthcare provider. If participants reported diagnoses and healthcare providers in the second assessment that differed from the first assessment, the second assessment was not considered for retest reliability assessments. Then we checked for floor- and ceiling effects as well as for the presence of central tendency bias, and excluded respective cases.

In the remaining sample of 165 participants who completed the first assessment all individual item responses were analyzed with respect to their psychometric properties according to the principles of classical test theory. We analyzed the item difficulties and skewness across all 168 items. In addition, we checked for items that showed high correlations with social desirability, in order to identify inadequate items (i.e. items with restricted validity that reflect a high tendency towards socially desirable responses). Then, we selected the respective German items that constitute the original HEAL-SF. Based on this short forms of HEAL-D, we calculated the internal consistency (Cronbach’s α) and the discrimination (corrected item-total-correlation) per scale, and the skewness for each of the 6 HEAL scales, as well as the correlation of the scales with social desirability. We assessed the comparability between the German short and long versions by correlating the scale means of both versions. Finally, we tested the retest-reliability by correlating the item means, as well as the scale means between the first and second assessment using the data from 115 participants who completed both assessments.

Next, confirmatory factor analyses (CFA) were carried out with the HEAL-D-SF in the sample of 165 participants who completed the first assessment, using R, “lavaan” package (37). Maximum likelihood estimation was used, with full information maximum likelihood for the missing data. Standardized latent factors were standardized, allowing free estimation of all factor loadings. Following recommendations of Kline (38), Hu and Bentler (39), and McDonald (40), four fit indices were used to examine the data-model fit of the CFA: (a) the chi-square test statistic, (b) the root-mean-square error of approximation (RMSEA), (c) the standardized root-mean-square residual (SRMR), and (d) the comparative fit index (CFI). As the chi-square test statistic is known to be influenced by sample size, model fit was assessed by determining whether the observed chi-square value divided by df (*χ*^2^/df) was smaller than three (41). Regarding RMSEA, a cutoff value of 0.06 or lower was required for a relatively good fit (39), whereas values between 0.08–0.061 indicate a reasonable model fit (42). For the SRMR, Hu and Bentler (39) recommended a value close to 0.08 or lower. Finally, the CFI has a cutoff value close to 0.95 (39). Regarding differences between the models of invariance, changes in CFI of 0.01 or less reveal that the invariance hypothesis should not be rejected (43). Given that the interpretation of model fit in CFA is not without some degree of controversy, all these indices of fit were used, and evaluation was based on convergence among findings (39, 44).

Modification indices informed how the model fit would have changed if we would have added new parameters to the model. However, since the CFA model was not exploratory, we decided to only specify a particular modification of the model if this was theoretically justifiable (45).

All analyses were conducted using the open-source software environment R (version 3.3.1; 46). We assumed statistical significance if the 2-sided *p* was smaller than 0.05.

## Results

### Socio-Demographic and Clinical Sample Characteristics

Two hundred forty four participants provided informed consent and started the online survey. Of those, 59 had to be excluded because they submitted an empty survey or did not complete at least one of the HEAL-D scales. In 32 cases we had to omit the second assessment, because they provided insufficient data for the retest reliability calculations, and in 10 cases we did not use the second assessment, because the healthcare provider differed between the first and second assessment. No single case had to be excluded because of occurring floor or ceiling effects or central tendency bias. The final sample, that completed the first assessment, and that was used for most analyses, consisted of 165 participants (86.7% female). The median age was 22 years (ranging from 19 to 48 years). Ninety eight percent of participants had at least a high school degree. The included participants reported a variety of reasons for seeking treatment. The most prevalent health complaints in our sample were affective, emotional, or behavioral problems (including depression, posttraumatic stress disorder, anxiety disorders, bipolar disorder, attention deficit and hyperactivity disorder, and anorexia mentioned by 33 participants) followed by pain (mentioned by 31 participants). Ten participants referred to check-ups (e.g. yearly check-up at the dentist). Two authors independently classified the mentioned health issues as chronic, acute, or unclear. In the chronic category chronic headaches, migraines, anxiety disorders, depression, allergies, and asthma were mentioned most often. Less frequently mentioned were chronic infections, irritable bowel syndrome, neurodermatitis, and chronic orthopedic dysfunctions including scoliosis and instability of joints. We rated health issues as chronic in 85 cases (52%). In 32 cases (19%) we rated the mentioned problems as acute. In this category most participants referred to accidents, surgeries, or check-ups. But also dental issues were rated as acute. In the unclear category (48 cases; 29%) we included pain-related issues (e.g. headaches and back pain that were not described as chronic), sleep problems, premenstrual and menstrual complaints, deficiency symptoms, and problems with the digestive system that were neither explicitly described as a particular syndrome nor as chronic. **Table 1** summarizes the main characteristics of the study sample.

**Table 1:** Selected characteristics of the included sample.

	1st assessment	2nd assessment
N total	165	115
Gender		
Female (%)	143 (86.7)	100 (87)
Male (%)	21 (12.7)	15 (13)
Other (%)	1 (0.6)	0
Mean age (range)	22.90 (19–48)	22.56 (19–45)
Education		
University degree	35	20
High school	127	93
Other	3	2
Religion		
Buddhism	2	–
Christianity	91	–
Hinduism	2	–
Islam	3	–
No religion	67	–
Treatment provider		
Acupuncturist	1	0
Dentist	3	3
Dermatologist	1	1
General practitioner	89	60
Gynecologist	3	1
Massage therapist	1	0
Neurologist	1	0
Non-medical practitioner	4	4
Occupational therapist	1	1
Physiotherapist	29	22
Psychotherapist	30	21
Psychologist	1	0
Psychiatrist	1	1

–, not assessed.

### Item and (Sub-)Scale Analyses

#### Item Characteristics of HEAL-D-SF

The items for the short-forms were selected in parallel to the original HEAL-SF. **Table 2** displays the item characteristics of the HEAL-D-SF.

**Table 2:** Item characteristics of HEAL-D-SF based on the 165 participants who completed the first assessment.

Items (English Original)	Mean (SD)	Discrimi-nation^a^	Skewness	Difficulty	Correlation with BIDR- IM (*p* -value)	Standardized factor loading^a,b^ (SE)
**Patient Provider Connection (PPC)**						
Ich bin mit meinem Behandler zufrieden. (I am satisfied with my healthcare provider.)	3.01 (0.99)	0.74	-0.76	0.75	**0.38 (< 0.001)**	0.76 (0.07)
Ich vertraue meinem Behandler. (I trust my healthcare provider.)	3.19 (0.88)	0.71	-0.98	0.8	**0.32 (< 0.001)**	0.67 (0.06)
Mein Behandler geht auf meine individuellen Bedürfnisse ein. (My healthcare provider pays attention to my individual needs.)	2.98 (0.90)	0.51	-0.66	0.75	**0.18 (0.02)**	0.47 (0.07)
Mein Behandler informiert mich ausreichend. (My healthcare provider gives me enough information.)	3.18 (0.77)	0.58	-0.86	0.79	**0.28 (< 0.001)**	0.55 (0.06)
Mein Behandler respektiert mich. (My healthcare provider respects me.)	2.79 (1.46)	0.79	-0.86	0.7	**0.58 (< 0.001)**	0.88 (0.09)
Ich habe das Gefühl, mein Behandler versteht mich. (I feel my healthcare provider understands me.)	2.5 (1.37)	0.81	-0.61	0.62	**0.49 (< 0.001)**	0.89 (0.08)
Mein Behandler unterstützt und ermutigt mich. (My healthcare provider gives me support and encouragement.)	2.42 (1.40)	0.81	-0.59	0.61	**0.49 (< 0.001)**	0.88 (0.09)
**Health Care Environment (HCE)**						
Das Personal ist respektvoll. (The staff was respectful.)	2.68 (1.47)	0.63	-0.7	0.67	**0.56 (< 0.001)**	0.91 (0.09)
Das Personal ist freundlich. (The staff was friendly.)	3.2 (0.88)	0.71	-1.15	0.8	**0.24 (0.002)**	0.57 (0.006)
Das Personal ist hilfsbereit. (The staff was helpful.)	3.31 (0.79)	0.66	-1.26	0.83	0.15 (0.06)	0.46 (0.06)
Die Versorgungsabläufe am Ort meiner Behandlung sind gut organisiert. (My care was well organized.)	2.27 (1.66)	0.70	-0.39	0.57	**0.59 (< 0.001)**	0.95 (0.10)
Die Räumlichkeiten ermöglichen den Schutz meiner Privatsphäre. (The healthcare provider's office respected my privacy.)	3.11 (0.91)	0.40	-0.84	0.78	0.22 (0.005)	0.26 (0.07)
Der Wartebereich ist ansprechend. (The waiting area was comfortable.)	2.81 (0.97)	0.30	-0.79	0.7	-0.03 (0.70)	0.12 (0.08)
**Treatment Expectancy (TE)**						
Ich habe Zuversicht in diese Behandlung. (I am confident in this treatment.)	3.09 (0.84)	0.73	-0.78	0.77	-0.03 (0.70)	0.85 (0.06)
Diese Behandlung wird erfolgreich sein. (This treatment will be successful.)	3.01 (0.88)	0.69	-0.71	0.75	**-0.20 (0.01)**	0.78 (0.06)
Ich fühle mich mit dieser Behandlung wohl. (I feel good about this treatment.)	3.28 (0.82)	0.63	-1.21	0.82	**-0.21 (0.008)**	0.76 (0.06)
Ich erwarte von dieser Behandlung gute Ergebnisse. (I expect good outcomes from this treatment.)	2.99 (1.00)	0.65	-0.92	0.75	0.03 (0.70)	0.71 (0.07)
Diese Behandlung ist die richtige für mich. (This treatment is right for me.)	2.62 (1.38)	0.44	-0.7	0.65	**0.36 (< 0.001)**	0.45 (0.11)
Ich schätze diese Behandlung. (I value this treatment.)	3.13 (0.82)	0.69	-0.58	0.78	**0.16 (0.05)**	0.72 (0.06)
**Positive Outlook (PO)**						
Ich habe meinem Leben gegenüber ein positives Gefühl. (I feel positive about my life.)	2.93 (0.92)	0.57	-0.9	0.73	**0.19 (0.01)**	0.27 (0.07)
Ich sehe meiner Zukunft hoffnungsvoll entgegen. (I am hopeful about my future.)	2.72 (1.02)	0.55	-0.59	0.68	**0.36 (< 0.001)**	0.50 (0.08)
Meine Zukunft sieht gut aus. (My future looks good.)	2.22 (1.4)	0.53	-0.44	0.56	**0.63 (< 0.001)**	1.00 (0.08)
Ich bin mit meinem Leben zufrieden. (I am satisfied with my life.)	3.11 (0.9)	0.34	-1.02	0.78	-0.12 (0.13)	0.02 (0.07)
Ich fühle mich selbstsicher. (I feel confident about myself.)	1.81 (1.26)	0.59	-0.2	0.45	**0.45 (< 0.001)**	0.80 (0.08)
Ich habe das Gefühl, dass ich mit meinen Problemen umgehen kann. (I feel I can cope with my problems.)	2.79 (0.88)	0.31	-0.74	0.7	-0.04 (0.63)	-0.01 (0.07)
**Spirituality (SP)**						
Spirituelle Glaubensinhalte geben meinem Leben Bedeutung. (Spiritual beliefs give meaning to my life.)	1.28 (1.44)	0.87	0.59	0.32	**-0.49 (< 0.001)**	0.91 (0.09)
Spirituelle Glaubensinhalte geben mir Hoffnung. (Spiritual beliefs give me hope.)	1.45 (1.56)	0.90	0.45	0.36	**-0.48 (< 0.001)**	0.94 (0.09)
Ich finde Trost in meinem Glauben. (I find comfort in my faith.)	1.49 (1.6)	0.91	0.46	0.37	**-0.45 (< 0.001)**	0.94 (0.09)
Meine Spiritualität gibt mir innere Stärke. (My spirituality gives me inner strength.)	1.76 (1.48)	0.84	0.16	0.44	**-0.41 (< 0.001)**	0.87 (0.09)
Beten ist ein bedeutsamer Teil meines Lebens. (Prayer is a meaningful part of my life.)	0.6 (1.08)	0.42	1.73	0.15	0.02 (0.84)	0.41 (0.08)
Ich fühle mich von einer höheren Macht unterstützt. (I feel supported by a higher power.)	1.52 (1.45)	0.88	0.28	0.38	**-0.39 (< 0.001)**	0.90 (0.09)
**Complementary and Alternative Medicine (CAM)**						
KAM ist wirksam. (CAM is effective.)	2.62 (0.90)	0.68	-0.42	0.65	**-0.15 (0.05)**	0.85 (0.06)
Ich bevorzuge KAM gegenüber Schulmedizin. (I prefer CAM over conventional medicine.)	2.2 (1.32)	0.47	-0.23	0.55	**-0.35 (< 0.001)**	0.80 (0.10)
Es ist wichtig, KAM gegenüber offen zu sein. (It is important to be open to CAM.)	2.38 (1.34)	0.27	-0.42	0.59	**0.51 (< 0.001)**	0.09 (0.12)
KAM kann zur Behandlung schwerer Krankheiten eingesetzt werden. (CAM can be used to treat serious illness.)	2.24 (1.08)	0.55	-0.16	0.56	-0.06 (0.42)	0.68 (0.08)
KAM kann gesundheitlichen Problemen vorbeugen. (CAM can prevent health problems.)	2.76 (1.06)	0.62	-0.74	0.69	-0.08 (0.29)	0.76 (0.08)
Ich bevorzuge natürliche Heilmittel. (I prefer natural remedies.)	1.68 (1.36)	0.41	0.26	0.42	**0.38 (< 0.001)**	0.24 (0.12)

^a^calculated per scale; ^b^standardized factor loadings from confirmatory factor analysis: HEAL-D-SF; SD, standard deviation; SE, standard error.

#### Characteristics of the HEAL-D-SF Scales and the BIDR Subscales

**Table 3** shows the relevant psychometric properties of the applied scales. The HEAL-D-SF scales showed acceptable to excellent internal consistencies between 0.74 and 0.93. The retest reliability ranged between 0.71 and 0.96. Five of the scales were significantly skewed (all *p* < 0.02).

**Table 3:** Psychometric Properties and Correlations of HEAL-D-SF with Impression Management, and HEAL-D (N = 165).

HEAL-D-SF scale	Mean (SD)	Cronbach’s α	Retest reliability	Skewness^a^ (*p*-value)	Correlation with BIDR-IM^b,^ ^c^ (*p*-value)	Correlation with HEAL-D^c^ (*p*-value)
Patient provider connection (CPP)	2.87 (0.89)	0.89	0.71	**-0.43 (< 0.001)**	**0.52 (< 0.001)**	**0.93 (< 0.001)**
Healthcare Environment (HCE)	2.89 (0.81)	0.79	0.88	**-0.57 (< 0.001)**	**0.48 (< 0.001)**	**0.90 (< 0.001)**
Treatment Expectancy (TE)	3.02 (0.72)	0.83	0.80	**-0.69 (< 0.001)**	0.06 (0.42)	**0.86 (< 0.001)**
Positive Outlook (PO)	2.60 (0.71)	0.74	0.76	**-0.15 (0.01)**	**0.43 (< 0.001)**	**0.66 (< 0.001)**
Spirituality (SP)	1.35 (1.25)	0.93	0.96	**0.36 (< 0.001)**	**-0.44 (< 0.001)**	**0.98 (< 0.001)**
Attitudes towards CAM (CAM)	2.31 (0.78)	0.74	0.85	-0.16 (0.15)	0.09 (0.23)	**1.0 (< 0.001)**

^a^significant deviations from normal distribution are printed in bold face; ^b^The adapted version of the BIDR-IM scale was used in which items with negative item-to-total correlations were deleted; ^c^ significant correlations are printed in bold face; SD, standard deviation.

The BIDR-SD showed an unacceptably low internal consistency (0.31), and the BIDR-IM showed a questionable internal consistency (0.61). As we found three items with negative discrimination among the BIDR items, we deleted those items and repeated the analyses using the BIDR subscales. In the adapted version the BIDR subscales’ internal consistency improved slightly with Cronbach’s α 0.54 for BIDR-SD and Cronbach’s α 0.65 for BIDR-IM. The retest reliability of the adapted BIDR scales was *r* = 0.67 SD and *r* = 0.82 for IM, and both subscales were significantly skewed (*p* = 0.01, and *p* = 0.009, respectively). Due to the poor reliability of the BIDR-SD subscale (even after adaption), we did not use this scale for further correlation analyses, and we used the adapted version of BIDR-IM for the following correlation analyses.

### Correlation Analyses

Four of the HEAL-D-SF scales showed significant correlations with BIDR-IM. The correlations between the short and long versions of HEAL-D were moderate to high ranging from *r* = 0.66 (positive outlook) to *r* = 0.98 (spirituality), indicating that the two versions are highly consistent. **Table 3** shows the respective correlation coefficients.

### Testing the Factor Structure of the HEAL-D-SF Scales

For our CFA the standardized factor loadings of most items were significant and most were larger than 0.4, except for the loading of five items (see **Table 2** for details). Nevertheless, the initial model fit of the German version of the HEAL-SF was not sufficiently satisfying [χ^2^: 2237.04; df: 614; *p* < 0.000; RMSEA: 0.13 with 90% CI (0.12, 0.13); RMR: 0.18, and CFI: 0.68] (**Table 4**). Modification indices found that specifying the presence of covariance for the error terms of one pair of items on the HCE factor, two pairs of items on the PO factor, and one pair of items on the CAM factor would significantly improve model fit (see **Table 4** for details). Given that each pair of items contained related content and the same factor, it was judged appropriate to adjust the model such that the error terms of these items were allowed to covary^1^. All indicators of model fit (**Table 4**) suggested that the adjusted model had a slightly better, but still non-acceptable fit with the data.

**Table 4:** Fit indices for the confirmatory factor analysis of HEAL-D-SF.

Model	*χ*^2^	df	*p*	*χ*^2^/df	RMSEA (90% CI)	SRMR	CFI
6 factor	2237.04	614	< 0.001	3.64	0.13 (0.12-0.13)	0.18	0.68
6 factor w/covaried error*	2005.85	610	< 0.001	3.29	0.12 (0.11-0.12)	0.18	0.73

*Model included specified covariance between error terms for the item “The staff was friendly” and the item “The staff was helpful” (both factor HCE); the item “I am satisfied with my life” and the item “I feel I can cope with my problems” (both factor PO); the item “I feel positive about my life” and the item “I am satisfied with my life” (both factor PO); as well as the item “It is important to be open to CAM” and the item “I prefer natural remedies” (both factor CAM). CFI, comparative fit index; df, degrees of freedom, RMSEA, root-mean-square error of approximation; SRMR, standardized root-mean- square residual.

## Discussion

### Main Findings

We set out to evaluate a parallel version of the HEAL-SF in German language. The HEAL items assess patients’ attitudes towards and perceptions of the so-called non-specific treatment components that have been shown to contribute to the effectiveness of inert treatments (e.g. sham interventions or placebos) but also to be responsible for a considerable amount of the effectiveness of pharmacological and psychotherapeutic treatments. The HEAL items have been developed applying rigorous methodology.

In the present study, the German HEAL items were used for the first time in an online survey in Switzerland. The six scales of HEAL-D-SF have demonstrated acceptable to excellent internal consistency and retest reliability, which indicate that the HEAL-D-SF scales are reliably applicable instruments. Most of the scales were skewed in our sample with most participants indicating high endorsement, except for the scale CAM. Given the well-organized and high-quality healthcare system in Switzerland, the skewness towards positive responses in the scales PCC, HCE, TE, and PO is no surprise. The scale SP was skewed towards negative responses, which may be explained by a poor relevance of spirituality in the selective sample of our study.

Using CFA, the six-factor structure of HEAL and HEAL-SF reported by Greco and colleagues (30) was partly confirmed using HEAL-D-SF: while factor loadings indicate a good fit of the items with the latent factors (i.e. scales) the overall model fit of the CFA was moderate to low. However, the model fit indices have been shown to largely depend on the sample size, which was comparably small in our study. By adjusting the original model following the highest modification index, which allows for covariation of error terms of several items, the model fit for the assessed fit indices slightly improved. Four items showed very low factor loadings as well as a low discrimination (HCE: “The waiting area was comfortable.”; PO: “I feel I can cope with my problems.” “I am satisfied with my life.”; CAM: “It is important to be open to CAM.”). If confirmed in future studies, these findings might indicate that the respective items represent different latent constructs compared with the other items of the respective scales.

Due to the poor psychometric quality of the BIDR scales, no conclusions are possible based on the significant correlations between the HEAL-D items and social desirability. In future studies the HEAL-D items need to be validated with additional reliable instruments.

### Relation to Relevant Previous Conceptual and Theoretical Work

HEAL and HEAL-SF have been constructed as a set of individual scales, which represent different aspects of treatments and of the according treatment context. The development of HEAL included a comprehensive overview of existing scales, and of expert and patient opinions. Although the authors of the original HEAL item banks did not explicitly relate the HEAL items to theoretical frameworks of non-specific factors, when relating the items to a prominent model of context factors proposed by Frank and Frank (47), the HEAL scales can be considered as operationalizations of the proposed factors: First, the scale HCE can be seen as including operational definitions of the *professional healthcare environment*. Second, the scale PPC can be seen as an operationalization of the *healing relationship*. Third, the scales TE, PO, SP, and CAM can be seen as contributing to ensuring that the advised and prescribed treatment (i.e. the *ritual*) and the *rationale* for this treatment are in line with patients’ expectations and attitudes and that they are thus acceptable for the patient as described by Budge and Wampold (48). Nevertheless, given the extreme variety of potentially relevant non-specific treatment aspects, the defined scales can only cover a part of all potentially relevant aspects, and additional operationalizations of the theoretical contextual factors are possible. In future, depending on the actual context, in which the HEAL items are to be administered, more scales tapping additional non-specific treatment components might be considered, and added to the HEAL item lists: For instance, items focusing on the provider’s empathy might be added to the HEAL item lists in future studies, as empathy has been demonstrated to be associated with treatment effects across different kinds of treatments, and is not explicitly addressed in the current HEAL item lists.

The possibility of assessing patients’ idiosyncratic perceptions of and attitudes towards treatment aspects besides the actively prescribed treatment components, can be seen as a further step to overcoming the invalid distinction between non-specific and specific treatment components and towards defining non-specific aspects of treatments as specific, as described for instance by Kaptchuk (49). The idea of “making the non-specifics specific” is not new: As early as 1973 Jefferson M. Fish proposed that that therapeutic processes have significant parallels to those taking place in faith-healing and placebo mechanisms in general (50). Along similar lines Frank characterized healing as a social influence process (47), and emphasized the relevance of the non-specific treatment components by presenting a contextual treatment model. More recently, Weinberger argued against using the term non-specific in the context of psychotherapeutic treatments: “I would prefer to say that some important factors may have not been operationalized well enough to be studied empirically; they have not yet been specified. Thus, they are non-specified, not non-specific. Contrary to the views of those questioning their scientific bona fides …, so-called non-specific effects are not ontologically non-specific. They are capable of being empirically specified.” (17). The outlined views on the relevance of “making the non-specifics specific” are also reflected by a recent feature in The British Medical Journal entitled “Social prescribing: coffee mornings, singing groups, and dance lessons on the NHS” (51), which outlines the idea to formalize physicians’ referrals of patients to community activities, and highlights the relevance of the entire healing context for clinical practice.

### Implications for Clinical Practice

In clinical practice, placebo effects, and thus non-specific treatment aspects, moderate and mediate treatment outcomes significantly. However, if healthcare providers are not particularly sensitive towards the relevance of the non-specific treatment aspects, issues associated with these treatment aspects are likely to remain undetected. If a given patient had for instance a low expectancy regarding the efficacy of a necessary standard treatment, the patient’s negative perceptions might have negative consequences with respect to the administration of or the adherence to the prescribed treatment, which might lead to a treatment failure. The low expectancy, however, might not appear to be relevant to the patient (and neither to the provider), and thus, might remain uncovered. In such a case, the administration of the HEAL items could help detecting the issue at hand. Then, the treatment provider could first take action in improving the patients’ outcome expectancy, before initiating the actual standard procedure.

As many of the non-specific treatment aspects, that impact treatment outcomes, are largely neglected in the context of standard treatment administration, the implementation of HEAL items in clinical practice might be seen as facilitating the detection of problematic aspects of a treatment, that are routed in the non-specific aspects of treatments. A deeper knowledge of patients’ idiosyncratic perceptions of and attitudes towards these would thus allow tailoring interventions in line with individual patients’ needs by facilitating an ethical and research-based conversation regarding what works in an intervention. This may in turn contribute to positive treatment expectations by providing a plausible treatment rationale.

It is important to note that we see the HEAL-D-SF as a flexible tool: Depending on the context of implementation, different scales may be of greater importance than others. For instance, the scale spirituality (SP) might help some patients to understand their symptoms within the context of their culture and religious beliefs. Concordantly, a recent meta-analysis revealed that treatments which are tailored to patients’ religious or spiritual beliefs are significantly more effective than no treatment or non-religious/spiritual psychotherapies in terms of psychological functioning (29). Along similar lines, a feature recently published in The British Medical Journal stated that there is a “high demand among the public for someone to talk to about spiritual matters in times of crisis” (52). The HEAL-SF spirituality scale can help to detect such needs in individual patients, and in turn the treatment provider and the patient can collaboratively discuss and decide, how the treatment can be adapted or complemented, in order to satisfy the patient’s need. Nevertheless, and to come back to the argument that a treatment should be credible and plausible, spirituality may not be relevant for every patient. We would therefore advise to judge from patient to patient, (or from context to context, respectively), whether the assessment of spirituality seems appropriate. The same holds true for the other scales of the HEAL-D-SF.

### Implications for Research

The development of HEAL-D-SF as a parallel version of the original HEAL-SF is of importance for healthcare research: The HEAL items offer the possibility to investigate the impact of non-specific treatment components across diverse interventions as well as across treatment contexts. The theory behind non-specific treatment components claims that these components have comparable effects across various interventions, treatment settings, and contexts. It would be interesting to test this assumption—e.g., to evaluate whether there is one factor which is the most reliable predictor for treatment success across cultures, populations, and treatment approaches using the parallel English and German versions of HEAL-SF. Importantly, these findings would be based on the patient’s own idiosyncratic views and assumptions, rather than relying on theoretical models or assumptions. Since HEAL-D-SF was developed in parallel to the existing HEAL-SF in English language, cross-cultural studies become possible in the future. Thus, a specific focus of future research projects can be the detection of similarities as well as dissimilarities in patients’ perception of the impact of non-specific treatment components on treatment outcomes—depending on patients’ cultural background.

### Limitations

Some limitations of the present study should be considered. First, and most important, the presented data are based on a comparably small sample, which may have negatively affected the overall model fit of the CFA, since fit indicators highly depend on the sample size. Hence, further studies are necessary that include larger samples in order to finally assess the factor structure of the German HEAL items. Second, our sample was heterogeneous with respect to the reported health conditions. While about half of the participants reported rather chronic conditions half of the participants reported rather acute conditions. It is possible, that the impact of the non-specific aspects of treatments on health outcomes varies depending on the chronicity of health conditions, and is, for instance, mediated by the intensity, frequency, and duration of the treatment. Along similar lines, non-specific aspects may have a greater impact in an ongoing treatment for a clinical condition when compared to a medical check-up. On the other hand, however, the HEAL item banks are considered to be condition-insensitive. Thus, the diversity of our sample with respect to the reported health complaints could be considered a strength of our study. Nevertheless, future studies should consider and test these possible moderators or mediators. Third, study participants were rather homogeneous with respect to educational level and age. It is possible that our findings will not generalize to populations with other socio-demographic characteristics. Therefore, future studies should include a broader range of study participants. Fourth, validation studies are necessary to test the convergent and discriminant validity as well as the prognostic value of the HEAL-D-SF items, including for instance comparisons with existing scales that assess non-specific factors more extendedly, and using longer item lists but also testing the prognostic value of HEAL items in predicting for instance health improvements or well-being in prospective studies. Fifth, the presented study relies on outpatient data assessed via online survey. For future studies it would be interesting to apply HEAL-D-SF in a clinical context.

### Conclusion

To conclude, we presented a German translation and a first evaluation of the HEAL items, that assess patients’ attitudes towards so-called non-specific treatment components. The German version (HEAL-D-SF) proved to be a reliable set of measures in an initial study. With six scales and six to seven items per scale the HEAL-D-SF are a parsimonious set of measures to assess the relevance of diverse non-specific treatment aspects. Especially when implemented in clinical practice, the shortness of HEAL-SF and HEAL-D-SF constitute a particular strength. But, before a possible application of HEAL-D items in clinical practice, additional validation studies are needed.

## Data Availability Statement

The datasets used for the analyses in this study are available from the corresponding author, upon reasonable request.

## Ethics Statement

This study was carried out in accordance with the recommendations of Ethikkommission Nordwest- und Zentralschweiz (Project-ID 2017-00870), with written informed consent from all subjects. All subjects gave written informed consent in accordance with the Declaration of Helsinki. The protocol was approved by the Ethikkommission Nordwest- und Zentralschweiz.

## Author Contributions

Study concept and design: HG, MG, CL. Acquisition, analysis or interpretation of data: SB, JG, HG, CL, DS. Drafting of the manuscript: HG, CL. Critical revision of the manuscript for important intellectual content: JG, HG, MG, CL. Statistical analyses: SB, HG, CL, DS. Study supervision: HG, CL.

## Funding

CL received funding for this project from the Swiss National Science Foundation (SNSF): P400PS_180730.

## Conflict of Interest

The authors declare that the research was conducted in the absence of any commercial or financial relationships that could be construed as a potential conflict of interest.

## Footnotes

1 Modification indices also suggested that specifying a covariance between the error terms of the items “*My healthcare provider pays attention to my individual needs*” and “*The staff was helpful*”, as well as of the items “*This treatment is right for me*” and “*It is important to be open to CAM*” would improve model fit. However, as each item pair was from separate scales and the item content was judged as non-similar, we felt it was not theoretically justifiable to specify these particular modifications of the model.

## References

[1] Wampold BE Minami T Tierney SC Baskin TW Bhati KS The placebo is powerful: estimating placebo effects in medicine and psychotherapy from randomized clinical trials. J Clin Psychol (2005) 61(7):835–54. 10.1002/jclp.20129 15827993

[2] Blease C The principle of parity: the ‘placebo effect’and physician communication. J Med Ethics (2012) 38(4):199–203. 10.1136/medethics-2011-100177 22048851

[3] Kelley JM Kraft-Todd G Schapira L Kossowsky J Riess H The influence of the patient-clinician relationship on healthcare outcomes: a systematic review and meta-analysis of randomized controlled trials. PloS One (2014) 9(4):e94207. 10.1371/journal.pone.0101191 24718585 PMC3981763

[4] Locher C Nascimento AF Kirsch I Kossowsky J Meyer A Gaab J Is the rationale more important than deception? A randomized controlled trial of open-label placebo analgesia. Pain (2017) 158(12):2320–8. 10.1097/j.pain.0000000000001012 28708766

[5] Rakel D Barrett B Zhang Z Hoeft T Chewning B Marchand L perception of empathy in the therapeutic encounter: effects on the common cold. Patient Educ Couns (2011) 85(3):390–7. 10.1016/j.pec.2011.01.009 PMC3107395 21300514

[6] Kaptchuk TJ Kelley JM Conboy LA Davis RB Kerr CE Jacobson EE Components of placebo effect: randomised controlled trial in patients with irritable bowel syndrome. BMJ (2008) 336(7651):999–1003. 10.1136/bmj.39524.439618.25 18390493 PMC2364862

[7] Rehman SU Nietert PJ Cope DW Kilpatrick AO What to wear today? Effect of doctor’s attire on the trust and confidence of patients. Am J Med (2005) 118(11):1279–86. 10.1016/j.amjmed.2005.04.026 16271913

[8] Paul GL Behavior modification research: design and tactics. In C. M. Franks (Ed.), Behavior therapy: Appraisal and status. New York: McGraw-Hill (1969).

[9] Borkovec TD Sibrava NJ Problems with the use of placebo conditions in psychotherapy research, suggested alternatives, and some strategies for the pursuit of the placebo phenomenon. J Clin Psychol (2005) 61:805–18. 10.1002/jclp.20127 15827991

[10] Kirsch I Placebo psychotherapy: synonym or oxymoron? J Clin Psychol (2005) 61(7):791–803. 10.1002/jclp.20126 15827992

[11] Schnurr PP The rocks and hard places in psychotherapy outcome research. J Trauma Stress (2007) 20(5):779–92. 10.1002/jts.20292 17955539

[12] Castelnuovo G Empirically supported treatments in psychotherapy: towards an evidence-based or evidence-biased psychology in clinical settings? Front In Psychol (2010) 1(27):1–10. 10.3389/fpsyg.2010.00027 PMC3153746 21833197

[13] Baskin TW Tierney SC Minami T Wampold BE Establishing specificity in psychotherapy: a meta-analysis of structural equivalence of placebo controls. J Consult Clin Psychol (2003) 71(6):973–9. 10.1037/0022-006X.71.6.973 14622072

[14] Frost ND Laska KM Wampold BE The evidence for present-centered therapy as a treatment for posttraumatic stress disorder. J Trauma Stress (2014) 27(1):1–8. 10.1002/jts.21881 24515534

[15] Gerger H Munder T Barth J Specific and nonspecific psychological interventions for ptsd symptoms: a meta-analysis with problem complexity as a moderator. J Clin Psychol (2014) 70(7):601–15. 10.1002/jclp.22059 24353221

[16] Khan A Brown WA Antidepressants versus placebo in major depression: an overview. World Psychiatry (2015) 14(3):294–300. 10.1002/wps.20241 26407778 PMC4592645

[17] Weinberger J Common factors are not so common and specific factors are not so specified: toward an inclusive integration of psychotherapy research. Psychotherapy (2014) 51(4):514–8. 10.1037/a0037092 25111381

[18] Mulder R Murray G Rucklidge J Common versus specific factors in psychotherapy: opening the black box. Lancet Psychiatry (2017) 4(12):953–62. 10.1016/S2215-0366(17)30100-1 28689019

[19] Cuijpers P Cristea IA What if a placebo effect explained all the activity of depression treatments? World Psychiatry: Off J World Psychiatr Assoc (2015) 14(3):310–1. 10.1002/wps.20249 PMC4592653 26407786

[20] Laska KM Gurman AS Wampold BE Expanding the lens of evidence-based practice in psychotherapy: a common factors perspective. Psychotherapy (2014) 51(4):467–81. 10.1037/a0034332 24377408

[21] Enck P Bingel U Schedlowski M Rief W The Placebo response in medicine: minimize, maximize or personalize? Nat Rev Drug Discovery (2013) 12(3):191–204. 10.1038/nrd3923 23449306

[22] Kalauokalani D Cherkin DC Sherman KJ Koepsell TD Deyo RA Lessons from a trial of acupuncture and massage for low back pain: patient expectations and treatment effects. Spine (2001) 26(13):1418–24. 10.1097/00007632-200107010-00005 11458142

[23] Constantino MJ Arnkoff DB Glass CR Ametrano RM Smith JZ Expectations. J Clin Psychol (2011) 67(2):184–92. 10.1002/jclp.20754 21128304

[24] Vase L Robinson ME Verne GN Price DD The contributions of suggestion, desire, and expectation to placebo effects in irritable bowel syndrome patients: an empirical investigation. Pain (2003) 105(1):17–25. 10.1016/S0304-3959(03)00073-3 14499416

[25] Wasan AD Kong J Pham L-D Kaptchuk TJ Edwards R Gollub RL The impact of placebo, psychopathology, and expectations on the response to acupuncture needling in patients with chronic low back pain. J Pain (2010) 11(6):555–63. 10.1016/j.jpain.2009.09.013 PMC2879448 20075014

[26] Clow KE Fischer AK Bryan DO Patient expectations of dental services. Market Health Serv (1995) 15(3):23. 10152791

[27] Birkhäuer J Gaab J Kossowsky J Hasler S Krummenacher P Werner C Trust in the healthcare professional and health outcome: a meta-analysis. PloS One (2017) 12(2):e0170988. 10.1371/journal.pone.0170988 28170443 PMC5295692

[28] Hyland ME Geraghty AW Joy OE Turner SI Spirituality predicts outcome independently of expectancy following flower essence self-treatment. J Psychosom Res (2006) 60(1):53–8. 10.1016/j.jpsychores.2005.06.073 16380310

[29] Captari LE Hook JN Hoyt W Davis DE McElroy-Heltzel SE Worthington EL Jr. Integrating clients’ religion and spirituality within psychotherapy: a comprehensive meta-analysis. J Clin Psychol (2018) 74(11):1938–51. 10.1002/jclp.22681 30221353

[30] Greco CM Yu L Johnston KL Dodds NE Morone NE Glick RM Measuring nonspecific factors in treatment: item banks that assess the healthcare experience and attitudes from the patient’s perspective. Qual Life Res (2016) 25(7):1625–34. 10.1007/s11136-015-1178-1 PMC4865446 26563249

[31] Cella D Riley W Stone A Rothrock N Reeve B Yount S the patient-reported outcomes measurement information system (promis) developed and tested its first wave of adult self-reported health outcome item banks: 2005–2008. J Clin Epidemiol (2010) 63(11):1179–94. 10.1016/j.jclinepi.2010.04.011 PMC2965562 20685078

[32] Cella D Yount S Rothrock N Gershon R Cook K Reeve B The patient-reported outcomes measurement information system (promis): progress of an nih roadmap cooperative group during its first two years. Med Care (2007) 45(5 Suppl 1):S3. 10.1097/01.mlr.0000258615.42478.55 PMC2829758 17443116

[33] Beaton DE Bombardier C Guillemin F Ferraz MB Guidelines for the process of cross-cultural adaptation of self-report measures. Spine (2000) 25(24):3186–91. 10.1097/00007632-200012150-00014 11124735

[34] Musch J Brockhaus R Bröder A Ein Inventar Zur Erfassung Von Zwei Faktoren Sozialer Erwünschtheit. Diagnostica (2002) 48(3):121–9. 10.1026//0012-1924.48.3.121

[35] Paulhus DL Balanced Inventory of Desirable Responding: Reference Manual for Bidr Version 6. Unpublished manuscript. University of British Columbia, Vancouver, Canada (1994).

[36] Schmitz C Limesurvey: An Open Source Survey Tool. LimeSurvey Project: Hamburg, Germany (2012).

[37] Rosseel Y Lavaan: An R Package for Structural Equation Modeling and More. Version 0.5–12 (Beta). J Stat Software (2012) 48(2):1–36.

[38] Kline RB Principles and Practice of Structural Equation Modeling. 4 ed. The Guilford Press: New York, NY (2015).

[39] Hu Lt Bentler PM Cutoff criteria for fit indexes in covariance structure analysis: conventional criteria versus new alternatives. Struct Equ Model: A Multidiscip J (1999) 6(1):1–55. 10.1080/10705519909540118

[40] McDonald R Test Theory: A Unified Treatment. Lawrence Erlbaum: Mahwah NJ (1999).

[41] Hallensleben N Spangenberg L Kapusta N Forkmann T Glaesmer H The German version of the interpersonal needs questionnaire (inq)–dimensionality, psychometric properties and population-based norms. J Affect Disord (2016) 195:191–8. 10.1016/j.jad.2016.01.045 26896813

[42] Schreiber JB Nora A Stage FK Barlow EA King J Reporting structural equation modeling and confirmatory factor analysis results: a review. J Educ Res (2006) 99(6):323–38. 10.3200/JOER.99.6.323-338

[43] Cheung G Rensvold R What constitutes significant differences in evaluating measurement invariance? 1999 *Conference of the Academy of Management*; 1999; Chicago.

[44] Tomarken AJ Waller NG Structural equation modeling: strengths, limitations, and misconceptions. Annu Revew Clin Psychol (2005) 1:31–5. 10.1146/annurev.clinpsy.1.102803.144239 17716081

[45] Vowles KE McCracken LM McLeod C Eccleston C The chronic pain acceptance questionnaire: confirmatory factor analysis and identification of patient subgroups. Pain (2008) 140(2):284–91. 10.1016/j.pain.2008.08.012 18824301

[46] Team R Core R: A Language and Environment for Statistical Computing. In: R Foundation for Statistical Computing. Vienna, Austria (2014).

[47] Frank JD Frank JB Persuasion and Healing: A Comparative Study of Psychotherapy. Johns Hopkins University Press: Baltimore, MD (1993).

[48] Budge SL Wampold BE The relationship: how it works. In: Gelo O Pritz A Rieken B, editors. Psychotherapy Research. Springer: Vienna (2015). p. 213–28.

[49] Kaptchuk TJ Placebo studies and ritual theory: a comparative analysis of navajo, acupuncture and biomedical healing. Philos Trans R Soc B: Biol Sci (2011) 366(1572):1849–58. 10.1098/rstb.2010.0385 PMC3130398 21576142

[50] Fish JM Placebo Therapy: A Practical Guide to Social Influence in Psychotherapy. Jossey-Bass: San Francisco (1973).

[51] Robinson A Social prescribing: coffee mornings, singing groups, and dance lessons on the Nhs. BMJ (2018) 363:k4857. 10.1136/bmj.k4857

[52] Hurley R Chaplaincy for the 21st Century, for People of all religions and none. BMJ (2018) 363:k5223. 10.1136/bmj.k5223

